# An examination of the association between forced sex history and reproductive coercion experiences among Black women attending STD clinics in Baltimore, MD, USA

**DOI:** 10.1186/s12978-023-01602-2

**Published:** 2023-05-15

**Authors:** Danielle M. Campbell, Marguerite B. Lucea, Andrea N. Cimino, Jacquelyn C. Campbell, Jamila K. Stockman

**Affiliations:** 1 Division of Infectious Diseases and Global Public Health, Department of Medicine, School of Medicine, University of California, San Diego, 9500 Gilman Drive, MC 0507, La Jolla, CA 92093-0507 USA; 2 School of Public Health, San Diego State University, San Diego, CA USA; 3 Division of Preventive and Social Medicine, School of Medicine, Charles R. Drew University of Medicine and Science, CA Los Angeles, USA; 4 Department of Nursing, Towson University, Towson, MD USA; 5 School of Nursing, Johns Hopkins University, Baltimore, MD USA

**Keywords:** Black women, Forced sex, Reproductive coercion victimization, Reproductive coercion perpetration, Physical violence, Psychological violence

## Abstract

**Background:**

Reproductive coercion victimization (RCV) is a significant public health issue that negatively affects women’s sexual and reproductive health outcomes. Less is known about reproductive coercion perpetration (RCP). Few studies have examined these phenomena among representative samples of Black women.

**Methods:**

Retrospective data of women (n = 298) attending STD clinics in Baltimore, MD were analyzed. We calculated lifetime and 12-month prevalence reports of reproductive coercion, and reported values stratified by forced sex history. Binomial logistic regression models were used to examine the association between forced sex history and RCV, accounting for other types of violence typologies.

**Results:**

Lifetime and past 12-month RCV and RCP prevalence were higher among women with forced sex experiences than their counterparts (Lifetime RCV: 46.9% versus 17.5%; past 12-month RCV: 19.4% versus 8.5%. Lifetime RCP: 24.5% versus 17%; past 12-month RCP: 13.3% versus 10.5%). Adjusted models, lifetime reproductive coercion: Women reporting forced sex had a 3.58 times higher odds of having had experienced RCV compared to women not reporting forced sex (AOR 3.58; 95% CI 2.00, 6.46). Women reporting forced sex had a 3.66 times higher odds of having ever experienced pregnancy coercion compared to their counterparts (AOR 3.66; 95% CI 1.93, 7.03) and 4.30 times higher odds of having ever experienced condom manipulation (AOR 4.30; 95% CI 2.15, 8.86). Adjusted models, past 12-month reproductive coercion: Women reporting forced sex had a 2.72 times higher odds of having had experienced past 12-month RCV compared to women not reporting forced sex (AOR 2.72; 95% CI 1.27, 5.91). Women reporting forced sex had a 3.25 times higher odds of having experienced past 12-month pregnancy coercion compared to their counterparts (AOR 3.25; 95% CI 1.38, 7.83) and 3.41 times higher odds of having experienced past 12-month condom manipulation (AOR 3.41; 95% CI 1.14, 10.98).

**Conclusions:**

Participants in our study reported high rates of RCV. Our novel exploration revealed significantly high rates of co-occurring forced sex experiences and RCV and initial prevalence report of RCP. Agencies have a unique opportunity to intervene by implementing screening protocols and referrals for supportive services. These findings may inform future intervention research efforts aimed at improving reproductive health outcomes among Black women.

## Background

Reproductive coercion victimization (RCV) encompasses verbal and physical acts perpetrated by a male partner to interfere with a woman’s reproductive autonomy [1–3]. RCV is contextualized within relationships where imbalances of ‘relational power,’ or one’s ability to act freely and make decisions that affect both individuals in a relationship, occur [4]. As a result, women experiencing reproductive coercion (RC) are less likely to exert control over their own sexual and reproductive freedoms.

Lifetime prevalence of RCV among women in the United States is estimated to be 8.4% but varies by racial and ethnic identity, age, and context. Non-Hispanic Black women are affected by RCV at higher rates than their Latina and Non-Hispanic White counterparts (22.9% versus 18.8% and 12.8%, respectively) [5, 6]. RCV prevalence among a sample of Black women residing in Baltimore, MD, USA, was as high as 37.8% [7]. Studies among college-aged women (18–24 years) revealed high RCV prevalence estimates of 25.3–29.6% [8]. Conversely, a cross-sectional study conducted among college students (18–25 years) in the Northeast USA demonstrated that approximately 8% of study participants had experiences with RCV [9]. Among Black women ages 18–24 in Atlanta, GA, USA, 23% reported ever having experienced any form of RC [10]. In addition, pregnancy coercion prevalence among Black women accessing sexual health services in family planning clinics has been noted as 25%, significantly higher than the general population [11].

### Mechanisms and health effects of RC

RC includes a range of behaviors including control of contraception, contraceptive sabotage, pregnancy pressure, and control of pregnancy outcome (e.g., unintended pregnancy). RC has been associated with condom manipulation (e.g., breaking or poking holes in condoms, non-consensual removal of condoms during sex), contraception interference (e.g., hiding or limiting access to oral contraception and/or removal of barrier contraception), and physical and psychological violence [1, 4, 6, 12].

RC has been associated with myriad negative health conditions, including both poor reproductive health as well as adverse mental health outcomes. Women experiencing RC report more unintended pregnancies, delayed access to prenatal healthcare, and low birthweight babies. Women experiencing RC are at increased vulnerability to sexually transmitted infections including HIV/AIDS [1, 11, 13]. Additionally, women experiencing RC have been more than twice as likely to exhibit PTSD symptoms [14]. Black women reporting experiences of RC have been nearly three times as likely to experience depression than women not reporting experiences of RC [7].

### Forced sex and RC

Forced sex (FS) is another prevalent concern for women and their health. One in six women report experiences of coerced or forced sex in their lifetime [15]. The health consequences of forced sex initiation and intimate partner violence on women’s health are well-documented in the literature, including many similar to those associated with RCV. The extent to which FS and RCV overlap is nascent in the extant literature. Further, the compendium of evidence focuses on RCV, and male-partner perpetrated physical and psychological control tactics. Even less is known about the relationship between women’s FS history and RC perpetration (RCP). Thus, our study objectives are to (1) describe the prevalence of self-reported RCV and RCP, including its subscales—pregnancy coercion and condom manipulation and (2) examine the association between forced sex (FS) history and RCV, accounting for other types of interpersonal violence (e.g., physical, psychological) among Black women attending STD clinics in Baltimore, MD, USA. We hypothesized that women with a history of FS would have a higher odds of lifetime and past-year RCV experiences including condom manipulation and pregnancy coercion. Given that little is known about RCP by women, our exploratory aim was to examine the association between FS history and self-reported RCP. To our knowledge, the current study is the first to simultaneously examine both RCV and RCP among Black women with and without histories of FS.

## Methods

### Study procedures

Secondary analyses were conducted using data from the ESSENCE Study, a retrospective cohort study that evaluated the impact of neighborhood-level characteristics of the built and social environment on FS, elucidating how FS and physiological factors influence behavioral mechanisms that increase risk for HIV acquisition. Data were collected between 2015 and 2018 among Black women (n = 305) accessing services at public health clinics in Baltimore, MD, USA [16]. Interested individuals who provided informed consent had to meet the following eligibility criteria: (a) assigned female at birth, (b) be between ages 18–44 years, (c) identify as Black or African American, (d) have a negative HIV test result at the time of enrollment, (e) report sex with a man in the past 6 months, and (f) report one or more of the following risk behaviors in the past year: two or more male sexual partners; or sex with a high-risk male partner (i.e., one who has injected drugs, is HIV seropositive, has sex with men, or has been incarcerated). Participants completed a one-time, audio computer-assisted self-interview (ACASI) [17] administered survey and received $10 for completing the screener, and $25 for completing the survey.

### Measures

*Reproductive Coercion* (RC, dependent variable; victimization and perpetration) was measured using items from the reproductive coercion scale (RCS) [18]. The 9-item measure assesses participants’ experiences with RCV across subdomains of pregnancy coercion and condom manipulation, using dichotomous (Yes/No) response options (Cronbach’s alpha = 0.89). Example items included, “Has any partner ever told you not to use contraception?” and “Has any partner ever told you he would leave you if you didn’t get pregnant?” RCP was assessed by asking 11 questions adapted from the RCS that focused on RC and abusive behaviors enacted by the participant (Cronbach’s alpha = 0.87). Example items included, “Have you ever told your partner you would leave him if he didn’t get you pregnant?” and “Have you ever told your partner you would have a baby with someone else if he didn’t get you pregnant?" A dichotomous variable was created such that if participants answered ‘yes’ to any one of the scale questions, they were coded as ‘yes’ in the analyses. Lifetime and past 12-month RCV and RCP experiences were assessed.

*Forced sex* (FS, primary independent variable) was defined as physical force (e.g., hit, held down, use of a weapon) or threats thereof by a male perpetrator since the age 18 years. Two questions were asked upon screening due to the study design, in which two groups of women were recruited: exposed (FS history) and unexposed. Unexposed participants reported no experiences of physical and/or sexual violence since the age of 18 years.

*Psychological violence* (independent variable) was measured using the Women’s Experience with Battering (WEB) scale [19]. The 10-item scale assesses perceived vulnerability to psychological danger by a current or recent physically or sexually abusive partner (Cronbach’s alpha = 0.99). Example items included, “He makes me feel like I have no control over my life, no power, no protection” and “He makes me feel unsafe even in my own home.” Likert responses were scored on a scale of 1–6, from agree strongly to disagree strongly, and ranged from 10 to 60. Participant responses were summed and a cutoff value of 20 was used to establish increased vulnerability to psychological violence [20]. Scores were then dichotomized such that a score of 20 or higher was coded as ‘yes’ for experiencing psychological violence.

*Physical violence* and *respondent-perpetrated physical violence* (independent variables) were measured using the physical assault scale and injury scale of the CTS-2 (Cronbach’s alpha = 0.99 and 0.99, respectively) [21]. First, frequency was coded into a scale variable ranging from 0 to 25, using the median value of each range. For example, “Once in the past year” was coded to 1, “Twice in the past year” was coded to 2, “3–5 times in the past year” was coded to 4, “6–10 times in the past year” was coded to 8, “11–20 times in the past year” was coded to 15, “More than 20 times in the past year” was coded to 25. Some of the items used simplified options of “never, once, a few times, many times, not in less than 12 months, but it did happen.” In such cases, “never” was coded to 0, “once” was coded to 1, “a few times” was coded to 3, “many times” was coded to 25, “not in less than 12 months, but it did happen” was coded to 0. Thus, physical violence victimization score (7 items) ranged from 0 to 175 and respondent perpetration (18 items) ranged from 0 to 450.

Sociodemographic covariates included age (years), sexual orientation (straight/gay, lesbian or bisexual), current employment status (unemployed/employed), current relationship status (in a relationship/not in a relationship), and lifetime pregnancy history (have ever been pregnant/never been pregnant).

### Statistical analysis

Statistical analyses were performed using R Studio version 1.3.1093–1 [22]. Participants were excluded by listwise deletion to only include those with complete data (n = 298) [23]. We reported frequencies and percentages for categorical variables, and medians and interquartile ranges for continuous variables. Analyses included descriptive statistics and binomial logistic regression.

The prevalence of each RC subscale (lifetime and 12-month) was calculated and stratified by FS history. Binomial logistic regression was conducted to examine the association between the primary independent variable, FS history, other independent variables psychological violence, physical violence victimization, and respondent-perpetrated physical violence, and dependent variables of RCV (including subscales pregnancy coercion and condom manipulation) and RCP. Unadjusted models included a single independent variable in each model. Adjusted logistic regression models were conducted to examine the association between FS history (primary independent variable) and lifetime and past 12-month RC experiences (victimization and perpetration; dependent variables). We accounted for psychological violence, physical violence victimization, and respondent-perpetrated physical violence, and sociodemographic characteristics of age, sexual orientation, current employment status, current relationship status, and lifetime pregnancy history. Both unadjusted and adjusted models were also constructed to examine the association between FS history and the subscales of RCV (pregnancy coercion and condom manipulation).

## Results

### Descriptive statistics

Participant (n = 298) sociodemographic characteristics are described in Table 1. Overall, median age was 26 years old (IQR: 22, 31), and the majority of women identified as heterosexual or straight (n = 252, 84.6%), were currently employed (n = 182, 61.1%), and had an annual individual income < $10,000 USD (n = 178, 59.7%). Only 27.2% were in a current relationship. Significant differences were observed by FS history for some of the sociodemographics. Women reporting FS were significantly older compared to women not reporting FS (median = 28; IQR: 24.3, 34 versus median = 25; IQR 21, 29; p < 0.001). A lower percentage of women reporting FS self-identified as heterosexual or straight compared to their counterparts, had at least some college education, and were currently employed. More women reporting FS reported an individual annual income less than < $10,000 USD compared to their counterparts.

**Table 1:** Sociodemographic characteristics of Black women in the ESSENCE study, Baltimore, MD, 2015–2018 (n = 298)

Characteristic	Overall (n = 298)	History of forced sex since age 18 (n = 98)^a^	No history of forced sex (n = 200)^a^	Test statistic	p-value
Sociodemographics					
Median age (IQR)	26 (22, 31)	28 (24.3, 34)	25 (21, 29)	W^c^ = 12,886	**< 0.001**
Sexual orientation				χ^2d^ = 11.352	**< 0.001**
Heterosexual or straight	252 (84.6)	73 (74.5)	179 (89.5)		
Gay, Lesbian or Bisexual	46 (15.4)	25 (25.5)	21 (10.5)		
Education				χ^2d^ = 6.651	**0.036**
< HS grad	52 (17.4)	25 (25.5)	27 (13.5)		
HS grad	98 (32.9)	30 (30.6)	68 (34.0)		
≥ Some college	148 (49.7)	43 (43.9)	105 (52.5)		
Employment				χ^2d^ = 7.532	**0.006**
Unemployed	116 (38.9)	49 (50)	67 (33.5)		
Employed	182 (61.1)	49 (50)	133 (66.5)		
Annual Individual income				χ^2d^ = 5.704	**0.058**
< $10,000 USD^b^	178 (59.7)	64 (65.3)	114 (57.0)		
$10,000–$29,999 USD^b^	95 (31.9)	31 (31.6)	64 (32.0)		
≥ $30,000 USD^b^	25 (8.4)	3 (3.1)	22 (11.0)		
Currently in a relationship	81 (27.2)	26 (26.5)	55 (27.5)	χ^2d^ = 0.031	0.860
Children under age 18 in household	151 (50.7)	56 (57.1)	95 (47.5)	χ^2d^ = 2.447	0.118
Median Lifetime # male sex partners (IQR)	5 (3, 7)	5 (4, 8)	4 (3, 7)	W^c^ = 7416.5	0.114
Pregnancy history	172 (57.7)	72 (73.5)	100 (50.0)	χ^2d^ = 14.845	**< 0.001**

Values that are significant are shown in bold

*IQR* interquartile range, *HS* high school, *USD* United States dollar

^a^Data are in the format n (%) unless otherwise indicated. Some values may be smaller than the actual value due to missing

^b^US dollar

^c^Wilcoxon-test for 2-sample comparison

^d^Chi-square test without Yate’s continuity correction

Prevalence reports of RCV, RCP, and each RC subscale for both lifetime and past 12-month periods are described in Table 2. RCV: In the overall sample, 27.2% women reported lifetime RCV and 12.1% reported past 12-month RCV. Prevalence reports were significantly higher (p < 0.001) on RCV composite scores (CS) and each RCV subscale (e.g., pregnancy coercion (PC), condom manipulation (CM)) among women reporting both lifetime (CS: 46.9%, PC: 34.7%, CM: 31.6%) and past 12-month RCV (CS: 19.4, PC: 15.3%, CM: 10.2%) versus those not reporting FS history. RCP: For the overall sample, prevalence reports of lifetime and past 12-month RCP were 19.5% and 11.4%, respectively. Women reporting FS history reported significantly more (p < 0.001) RCP during lifetime and past 12 month (24.5% and 19.4%, respectively) compared to women not reporting FS history (17.0% and 8.5%, respectively). The differences in prevalence carried across the subscales of RCP as well, with the exception of past 12-month CM.

**Table 2:** Reproductive coercion victimization and perpetration prevalence estimates among Black women in the ESSENCE Study, Baltimore, MD (n = 298)

	Overall n (%^1^) (n = 298)	Forced sex history n (%^1^) (n = 98)	No history of forced sex n (%^1^) (n = 200)	p-value^2^
Lifetime				
Reproductive Coercion Victimization*	81 (27.2)	**46 (46.9)**	35 (17.5)	**< 0.001**
** Pregnancy Coercion**	58 (19.5)	**34 (34.7)**	24 (12.0)	**< 0.001**
** Condom Manipulation**	47 (15.8)	**31 (31.6)**	16 (8.0)	**< 0.001**
Reproductive Coercion Perpetration*	58 (19.5)	24 (24.5)	34 (17.0)	0.125
** Pregnancy Coercion**	16 (5.4)	**9 (9.2)**	7 (3.5)	**0.041**
Condom Manipulation	54 (18.1)	21 (21.4)	33 (16.5)	0.299
Past 12-Month				
Reproductive Coercion Victimization*	36 (12.1)	**19 (19.4)**	17 (8.5)	**0.007**
** Pregnancy Coercion**	27 (9.1)	**15 (15.3)**	12 (6.0)	**0.009**
** Condom Manipulation**	16 (5.4)	**10 (10.2)**	6 (3.0)	**0.010**
Reproductive Coercion Perpetration*	34 (11.4)	13 (13.3)	21 (10.5)	0.480
** Pregnancy Coercion**	8 (2.7)	**5 (5.1)**	3 (1.5)	**< 0.001**
Condom Manipulation	31 (10.4)	10 (10.2)	21 (10.5)	0.937

Values that are significant are shown in bold

*Indicates composite value

^1^Percentage related to the total sample (i.e., shown as column)

^2^2-sample test for equality of proportions of each reproductive coercion between respondents with and without the forced sex history. The analysis is conducted without continuity correction

### Binomial logistic regression-forced sex history, psychological violence, physical violence, respondent-perpetrated physical violence

Table 3 describes the unadjusted and adjusted binomial logistic regression models constructed to assess the association between FS history, psychological violence, physical violence, and respondent-perpetrated physical violence and lifetime RCV and RCP. In the unadjusted models for lifetime RCV, FS, psychological violence, and respondent-perpetrated physical violence reached statistical significance for the overall composite scale, as well as the subscales of pregnancy coercion and condom manipulation. For RC perpetration, only respondent-perpetrated physical violence reached statistical significance (OR = 1.03, 95% CI: 1.01, 1.07).

**Table 3:** Regression models of forced sex on lifetime reproductive coercion experiences among Black women, Baltimore, MD, 2015–2018 (n = 298)

	RCV Overall OR (95% CI)	Unadjusted Models		RCP OR (95% CI)	Adjusted Models^1^			RCP AOR (95% CI)
		Pregnancy Coercion RCV OR (95% CI)	Condom Manipulation RCV OR (95% CI)		RCV Overall AOR (95% CI)	Pregnancy Coercion RCV AOR (95% CI)	Condom Manipulation RCV AOR (95% CI)	
Forced Sex History	4.35*** (2.55, 7.50)	4.05*** (2.25, 7.42)	5.52*** (2.88, 10.96)	1.65 (0.91, 2.97)	3.58*** (2.00, 6.46)	3.66*** (1.93, 7.03)	4.30*** (2.15, 8.86)	1.74 (0.92, 3.27)
Psychological violence victimization	3.87*** (2.43, 6.31)	5.26*** (3.23, 8.69)	2.39*** (1.42, 3.96)	1.56 (0.92, 2.56)	3.42*** (2.08, 5.72)	4.93*** (2.94, 8.43)	1.99* (1.15, 3.38)	1.56 (0.91, 2.62)
Physical violence victimization	**1.03 (1.00, 1.07)**	**1.03 (1.00, 1.07)**	1.02 (0.99, 1.05)	1.01 (0.99, 1.04)	**1.02 (1.00, 1.05)**	**1.02 (1.00, 1.06)**	1.01 (0.99, 1.04)	1.01 (0.99, 1.04)
Respondent -perpetrated physical violence	1.19*** (1.09, 1.33)	1.04* (1.01, 1.09)	1.03* (1.01, 1.07)	1.03* (1.01, 1.05)	1.17** (1.07, 1.31)	1.03* (1.01, 1.08)	1.03* (1.01, 1.06)	1.03* (1.01, 1.05)

OR and CI values that are significant are shown in bold

^1^Adjusted for age, sexual orientation, current employment, relationship status, and lifetime pregnancy history

RCV- Reproductive Coercion Victimization OR, odds ratio; CI, confidence interval; AOR, adjusted odds ratio

RCP- Reproductive Coercion Perpetration Significance levels: ***< 0.001, **< 0.01, *< 0.05

Accounting for psychological violence, physical violence victimization, respondent-perpetrated physical violence, and sociodemographics, women reporting FS had a 3.58 times higher odds of having had experienced RCV compared to women not reporting FS (AOR 3.58; 95% CI 2.00, 6.46). Specific to the subscales, women reporting FS had a 4.30 times higher odds of having ever experienced condom manipulation (AOR 4.30, 95% CI 2.15, 8.86) and a 3.66 times higher odds of having ever experienced pregnancy coercion (AOR 3.66, 95% CI 1.93, 7.03) compared to their counterparts. In the adjusted model, forced sex retained its non-significant association with lifetime RCP.

Table 4 describes the unadjusted and adjusted binomial logistic regression models constructed to assess the association between the key independent variables and past-12-month RCV and RCP. In the unadjusted models, FS history, psychological violence, and respondent-perpetrated physical violence reached statistical significance for the overall composite RCV scale and both subscales. For RCP, only respondent-perpetrated physical violence was significantly associated in the unadjusted models (OR = 1.02, 95% CI 1.001, 1.0).

**Table 4:** Regression models of forced sex on past 12-month reproductive coercion experiences among Black women, Baltimore, MD, 2015–2018 (n = 298)

	RCV Overall OR (95% CI)	Unadjusted Models		RCP OR (95% CI)	Adjusted Models^1^			RCP AOR (95% CI)
		Pregnancy Coercion RCV OR (95% CI)	Condom Manipulation RCV OR (95% CI)		RCV Overall AOR (95% CI)	Pregnancy Coercion RCV AOR (95% CI)	Condom Manipulation RCV AOR (95% CI)	
Forced sex history	2.69** (1.33, 5.49)	2.94** (1.32, 6.66)	3.81* (1.37, 11.49)	1.65 (0.91, 2.97)	2.72* (1.27, 5.91)	3.25** (1.38, 7.83)	3.41* (1.14, 10.98)	1.63 (0.73, 3.57)
Psychological violence victimization	4.58*** (2.68, 7.90)	6.66*** (3.64, 12.61)	3.11** (1.45, 6.52)	1.56 (0.92, 2.56)	4.65*** (2.60, 8.48)	7.24*** (3.76, 14.61)	2.67* (1.20, 5.80)	1.26 (0.60, 2.43)
Physical violence victimization	**1.03 (1.00, 1.06)**	**1.03 (1.00, 1.06)**	1.01 (0.97, 1.04)	1.01 (0.99, 1.04)	1.02* (1.00, 1.06)	1.03* (1.00, 1.06)	1.01 (0.96, 1.04)	1.02 (0.99, 1.04)
Respondent perpetrated physical violence	1.05* (1.02, 1.11)	1.03** (1.01, 1.06)	1.03** (1.01, 1.05)	1.02* (1.00, 1.04)	1.05** (1.02, 1.09)	1.03** (1.01, 1.06)	1.03* (1.01, 1.05)	1.02* (1.00, 1.05)

^1^Adjusted for age, sexual orientation, current employment, relationship status, and lifetime pregnancy history

RCV- Reproductive Coercion Victimization OR, odds ratio; CI, confidence interval; AOR, adjusted odds ratio

RCP- Reproductive Coercion Perpetration Significance levels: ***< 0.001, **< 0.01, *< 0.05

OR and CI values that are significant are shown in bold

In the adjusted models, women reporting FS were nearly three times more likely to have experienced RCV in the past 12-months compared to women not reporting FS (AOR = 2.72, 95% CI 1.27, 5.91). Specific to the subscales, women reporting FS had a 3.25 and 3.41 higher odds of experiencing past 12-month pregnancy coercion and past 12-month condom manipulation, respectively, compared to women not reporting FS. Forced sex was not significantly associated with RCP.

Of note, when accounting for FS history and other independent variables in the adjusted models, psychological violence victimization remained significantly associated with higher odds for both lifetime and past 12-month RCV. The highest odds was for pregnancy coercion (Lifetime RCV: AOR 4.93; 95% CI 2.94, 8.43; Past 12-month RCV: AOR 7.24; 95% CI 3.76, 14.61). Physical violence victimization was significantly associated with higher past 12-month RCV (Overall: AOR 1.02 95% CI 1.001, 1.06; Pregnancy coercion: AOR 1.03; 95% CI 1.001, 1.06). Respondent-perpetrated physical violence was significantly associated with all forms of RCV and RCP for both lifetime and past 12-month.

## Discussion

RCV is a significant public health issue that disproportionately impacts Black women. Our study aimed to describe the prevalence of RCV and RCP and examine the association between FS history and lifetime and past 12-month RCV among Black women attending STD clinics in Baltimore, MD, USA. We also explored the relationship between FS history and RCP.

Overall lifetime prevalence reports of RCV among our sample of Black women (27.2%) were similar to those in previous studies (14.2% Basile [5] et al.; 37.1% Holliday et al. [24]). Our study was one of the first to explore RCV in the context of FS history among Black women. Women reporting a history of FS had a higher prevalence of both lifetime and past 12-month RCV, including pregnancy coercion and condom manipulation. Women reporting a history of FS also experienced an increased odds of both lifetime and past 12-month RCV including pregnancy coercion and condom manipulation. Our results supported our hypothesis that FS history would confer higher odds of experiencing RCV. Issues of agency, complicated by sexual trauma, may inhibit these women’s ability to negotiate control over their own bodies and choices.

Other violence typologies were also significantly associated with RCV. Psychological violence and physical violence victimization were prevalent among participants. Accounting for FS history and other variables, these remained significantly associated with lifetime overall RCV and specifically the subscale, lifetime pregnancy coercion RCV. The same significant relationships held when examining past 12-month RCV and the subset of past 12-month pregnancy coercion. Physical violence victimization was not significantly associated with the RCV subscale of condom manipulation in either of the models. This may indicate that, in the presence of physical violence, pregnancy coercion may be the predominant mechanism of RCV. This is supported by the non-statistically significant ORs and AORs of condom manipulation RCV subscale for women experiencing physical violence. We were not able to ascertain in this analysis the context surrounding lifetime and past 12-month RCV and specifically pregnancy coercion among women experiencing physical violence, but it could be hypothesized that, in the reconciliation and honeymoon phases of physical violence, the perpetrator may attempt to coerce the woman into pregnancy. This is certainly an area for further exploration to ascertain the temporality of the coercion type.

Of note, we were not able to ascertain if the presence of FS history and other aspects of violence had a compounding effect on RCV experience. Yet, given the similarities of the health consequences related to FS history, RCV, and other violence experiences separately, the co-occurrence is also important to note and explore further.

Our study is the first to examine RCP among Black women with and without FS histories. While RCP was more prevalent among women with FS histories, FS was not significantly associated with either lifetime or past 12-month RCP. We also did not observe any statistical significance when accounting for other violence typologies except respondent perpetrated physical violence. While we did not anticipate that women with RCV experiences would also be perpetrators of physical violence themselves, we were unable to establish temporality or violence typology due to instrument measurement limitations. For example, respondent-perpetrated physical violence may or may not have been initiated as a form of defense during a violent attack or unprovoked. This unexpected finding demonstrates the need for further exploration of the association between these variables.

Notwithstanding, our study faced several methodological limitations that may affect the generalizability of our findings. Our sample size was smaller than other RCV studies [5, 25]. However, few existing studies included representative numbers of Black women. Because we conducted a cross-sectional study, temporality of any associations cannot be determined [26]. Retrospective cross-sectional cohort studies are prone to participant recall bias [27]. Participants had to recall from memory the frequency of certain events or behaviors some of which could have induced an unwelcomed trauma response and lost memory [28]. Participants were recruited by non-probabilistic convenience sample among women in waiting rooms of two Baltimore City STD clinics. Therefore, our findings are not generalizable to Black women who do not seek STD services nor women of other racial and ethnic groups [29].

Despite the noted limitations, our study had numerous strengths. Our study sample was underserved and experienced high prevalence of violence and associated physical health outcomes including sexually transmitted infections, which demonstrates the need for our study findings to promote targeted sexual and reproductive health interventions. The exploratory investigation of RCP marks initial prevalence data among women, more specifically, Black women. Of importance, the inclusion of psychological violence victimization, an understudied form of violence, as a covariate provided a more robust assessment of the isolated effects of FS on RCV and RCP, as well as the independent effects of psychological violence victimization on RCV and RCP.

## Conclusion

The high rates of RCV and FS among Black women in our study calls attention to the critical need for trauma-informed care in the STD clinic setting. Sexual health clinics have a unique opportunity to screen women for all forms of violence, including RC and provide comprehensive health services including supportive referrals to ancillary support services. Since trauma-informed, intimate partner violence/RC screening and education has been shown to demonstrate promise in early violence detection and safety promotion among women experiencing intimate partner violence in family planning clinics [30, 31], it is critical to integrate these strategies in STD clinics. Our study findings may inform the development of evidence-based interventions aimed at improving reproductive and sexual health outcomes among Black women.

## Data Availability

The datasets generated and/or analyzed during the current study are available from the corresponding author on reasonable request.
